# Neural Differentiation Modulates the Vertebrate Brain Specific Splicing Program

**DOI:** 10.1371/journal.pone.0125998

**Published:** 2015-05-19

**Authors:** Alicia Madgwick, Philippe Fort, Peter S. Hanson, Philippe Thibault, Marie-Claude Gaudreau, Georges Lutfalla, Tarik Möröy, Sherif Abou Elela, Bill Chaudhry, David J. Elliott, Christopher M. Morris, Julian P. Venables

**Affiliations:** 1 Institute of Genetic Medicine, Newcastle University, Central Parkway, Newcastle NE1 3BZ, United Kingdom; 2 Centre Nationale de la Recherche Scientifique, CRBM-UMR5237, Université de Montpellier, Montpellier, France; 3 Medical Toxicology Centre, Wolfson Building, Newcastle University, Newcastle upon Tyne, United Kingdom; 4 Département de Microbiologie et d'Infectiologie, Faculté de Médecine et des Sciences de la Santé, Université de Sherbrooke, Québec, Canada; 5 Institut de recherches cliniques de Montréal, Université de Montréal, Québec, Canada; 6 Département de Microbiologie, Infectiologie, et Immunologie, Université de Montréal, Québec, Canada; 7 Dynamique des Interactions Membranaires Normales et Pathologiques, UMR 5235 CNRS-Université de Montpellier, Montpellier, France; International Centre for Genetic Engineering and Biotechnology, ITALY

## Abstract

Alternative splicing patterns are known to vary between tissues but these patterns have been found to be predominantly peculiar to one species or another, implying only a limited function in fundamental neural biology. Here we used high-throughput RT-PCR to monitor the expression pattern of all the annotated simple alternative splicing events (ASEs) in the Reference Sequence Database, in different mouse tissues and identified 93 brain-specific events that shift from one isoform to another (switch-like) between brain and other tissues. Consistent with an important function, regulation of a core set of 9 conserved switch-like ASEs is highly conserved, as they have the same pattern of tissue-specific splicing in all vertebrates tested: human, mouse and zebrafish. Several of these ASEs are embedded within genes that encode proteins associated with the neuronal microtubule network, and show a dramatic and concerted shift within a short time window of human neural stem cell differentiation. Similarly these exons are dynamically regulated in zebrafish development. These data demonstrate that although alternative splicing patterns often vary between species, there is nonetheless a core set of vertebrate brain-specific ASEs that are conserved between species and associated with neural differentiation.

## Introduction

How both complex and simple body plans (e.g. human and nematode) can be encoded by of the order of only 20,000 genes is a major conundrum in genome biology. One part of the solution to this is alternative splicing, a mechanism through which different RNAs and protein products can be made from a single gene by differential incorporation of regions of pre-mRNA. Alternative splicing is a key player in gene expression of complex organisms like mammals and in complex tissues like the brain as it increases transcriptome and thereby proteome diversity. For two decades, alternative splicing has been studied on a gene-by-gene basis. In the last fifteen years a significant amount of transcriptome-wide data from microarray and RNA-Seq experiments has increased the number of known alternative splice events (ASEs). Recent developments have brought to light that at least 80% of genes produce alternative mRNAs. Which of these ASEs are important for development is now a major question in the field. One route to answer this question is through evolutionary comparisons, on the assumption that natural selection has maintained the most important regulated ASEs.

High-throughput RT-PCR is a valuable method to identify splicing changes in many genes at once [1–3] and is an alternative to current methods including RNA-Seq [2,4]. We have recently used a cross-evolutionary approach coupled to high-throughput RT-PCR to identify the most conserved tissue-specific alternative exons, regulated across a variety of different organisms. Starting with a list of 40 human alternative exons we found one that was alternatively spliced in muscle across all deuterostomes, that is, including vertebrates, chordates, echinoderms and hemichordates [5]. The identification of highly conserved patterns of alternative splicing in a specific tissue within animals is important, since it reveals exceptions to other recent transcriptome-wide studies that show, using RNA-Seq, that alternative splicing is for the most part specific to individual species and not to tissues [6,7]. These results suggested that alternative splicing has only a minor role if any in formulating the vertebrate body plan.

Extreme changes, from near total exon inclusion to exon skipping, known as switch-like splicing, have been observed in human tissues, but not in all vertebrate orthologues [8]. Here we use high-throughput RT-PCR in a cross-evolutionary approach to identify a new panel of ASEs that are highly tissue-specific in vertebrates, and have identical splicing patterns in the brain of mammals and zebrafish. Using a stem cell model we find that these exons are a conserved component of alternative splicing changes that shift abruptly during neural stem cell differentiation, and might therefore be important for conserved features of vertebrate central nervous system development.

## Results

### RT-PCR mining of tissue-specific splicing reveals 93 switch-like brain-specific exons in mouse

**Fig 1:**
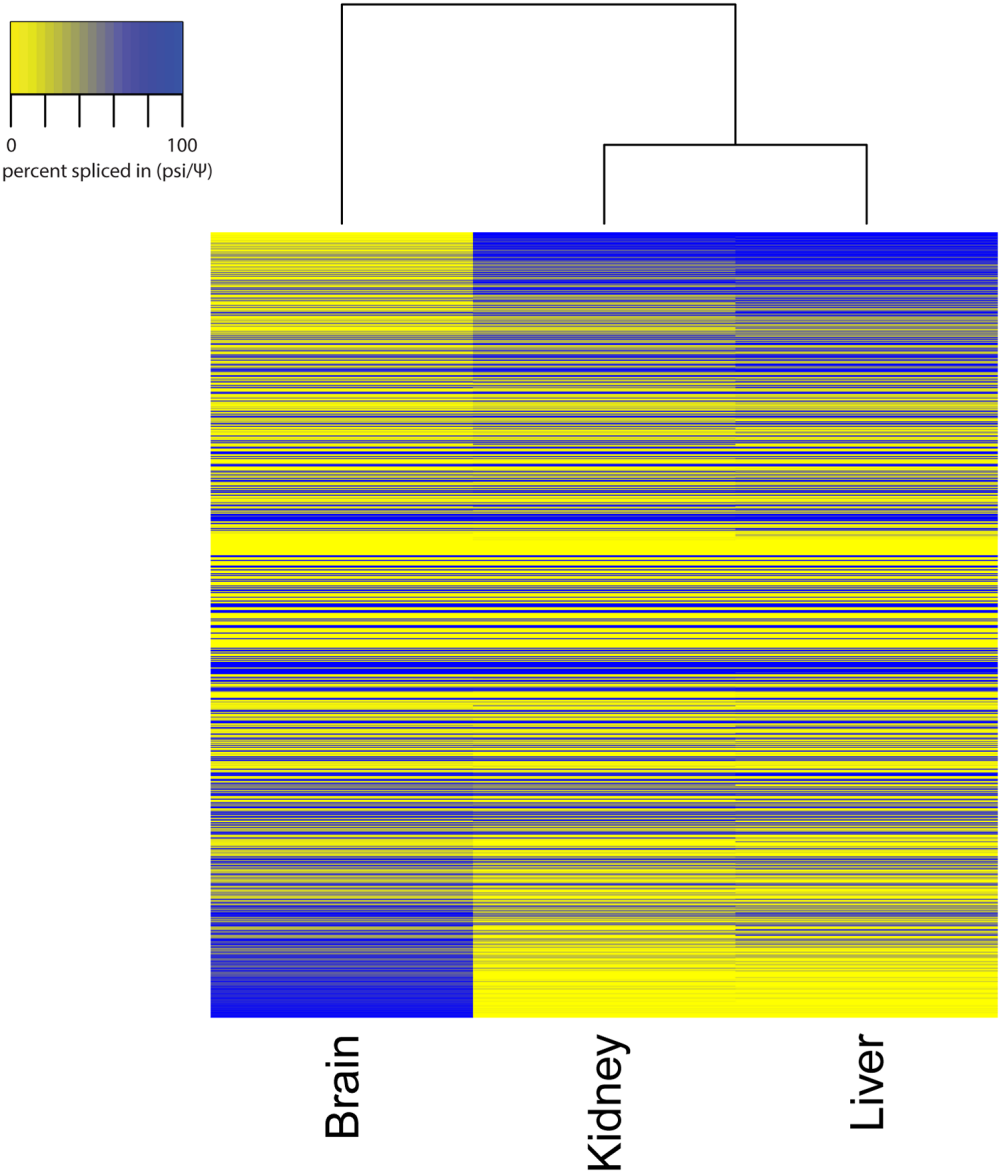
Genomewide screen for brain-specific ASEs in mouse. PCR primers were designed across all the simple ASEs in the mouse RefSeq collection [10] and RT-PCR was performed on three mouse tissue cDNA;. 809 PCRs gave good data; the psi values are shown for mouse brain, kidney and liver. The ASEs have been clustered according to the shift between brain and the two other tissues, indicated by the difference in psi values. 93 ASE psi values shifted more than 50% between brain and both of the other tissues (see the top and bottom of the heat map). Numerical data are given in S1 Table.

In order to first profile the extent of tissue-specific alternative splicing pattern variations in humans, we studied a panel of 47 [1] alternative splicing events (ASEs) across 6 human tissue cDNAs (Clontech, Mountain View, CA) by RT-PCR. These tissues were liver, kidney, lung, muscle, heart and brain (S1 Fig). As expected from previous high-throughput studies [7,9] these splicing events could be used to define three groups: brain with the most distinctive profile, muscle and heart with another profile and liver, kidney and lung as the third group. The largest difference in splicing profiles was between brain and liver/kidney. Therefore to select for important ASEs across the entire transcriptome we then compared splicing profiles of mouse brain, liver and kidney with a panel of 1329 (functionally random) ASEs composed of all the simple ASEs in the mouse RefSeq database [10]. After quality control, 809 PCRs produced robust data for all three samples. 70% of these (560/809) were simple cassette exons. The other events fell into three types: alternative 5’ sites (100), alternative 3’ sites (102) and more complex splice events (47). 93 ASEs showed a 50% shift in exon inclusion between brain and both other tissues (Fig 1; all splicing data is available numerically in S1 Table and all original electopherograms are at http://rnomics.med.usherbrooke.ca:3000/pcrreactiongroup/list/336). 84% of these brain-specific ASEs (78/93) were simple cassette exons, therefore conserved alternative splices are highly enriched in alternative exons compared to other types of alternative splicing (p = 0.00005 Fisher’s exact test).

### Nine alternative splice events are regulated in a switch-like brain-specific manner from man to zebrafish

**Fig 2:**
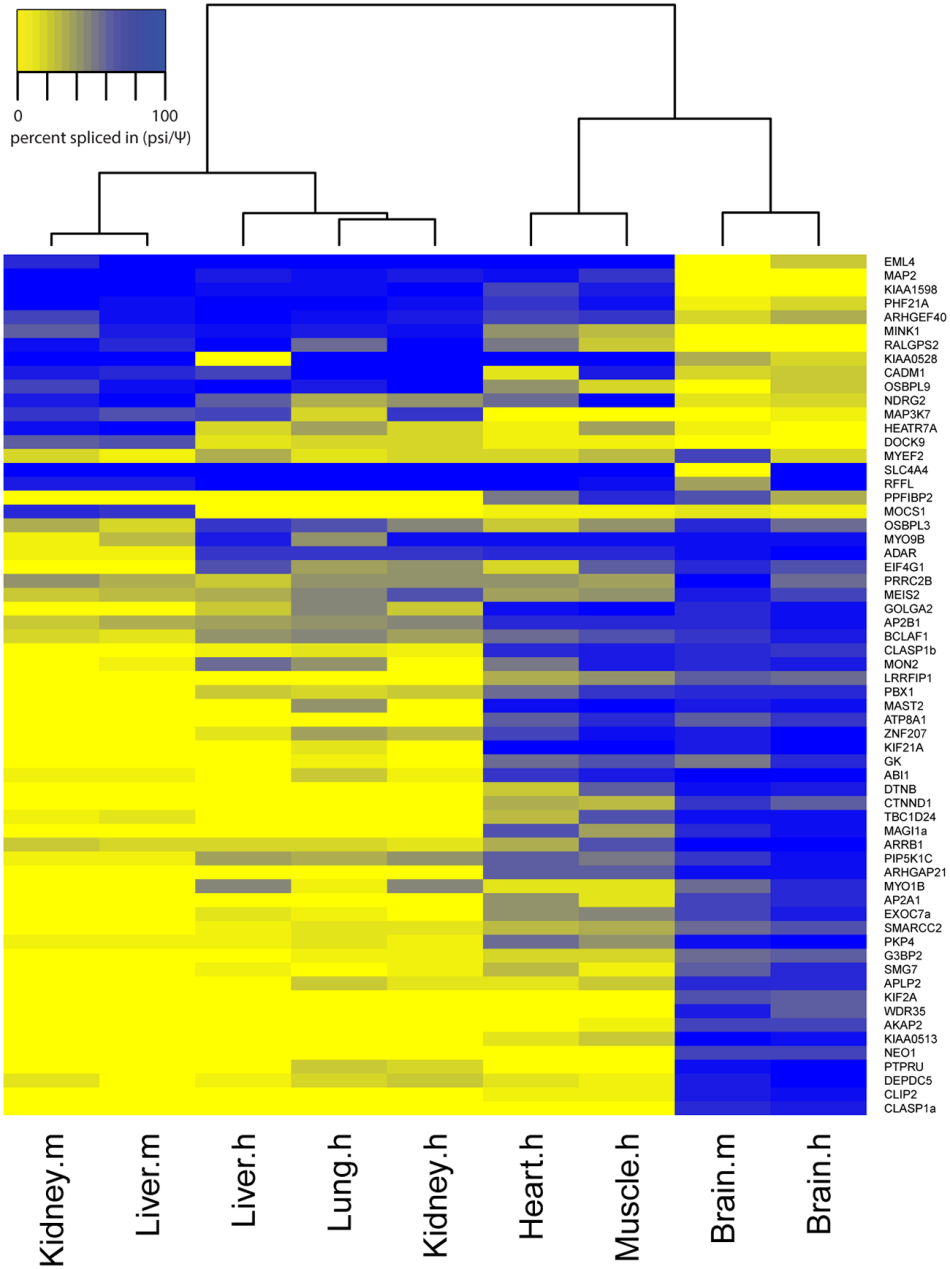
Cross-evolutionary screen for brain-specific ASEs found in human. Of the 93 mouse candidate ASEs for conserved brain-specificity, 89 had orthologous regions in human. Of these, 62 ASEs gave good data by PCR on 6 human tissue cDNAs: brain, heart, muscle, kidney, liver and lung. The psi values are shown in the heat map; the genes are clustered on both axes according to their psi values. X-axis clustering shows the brain, heart/muscle and liver/kidney/lung have 3 distinct splicing profiles. Y axis clustering groups the genes based on their psi values’ patterns across tissues; the brain-specific ASEs cluster at the bottom of the heat map but the clustering also distinguishes some ASEs in the centre that splice similarly in brain/heart/muscle as distinct from liver/kidney/lung.

**Fig 3:**
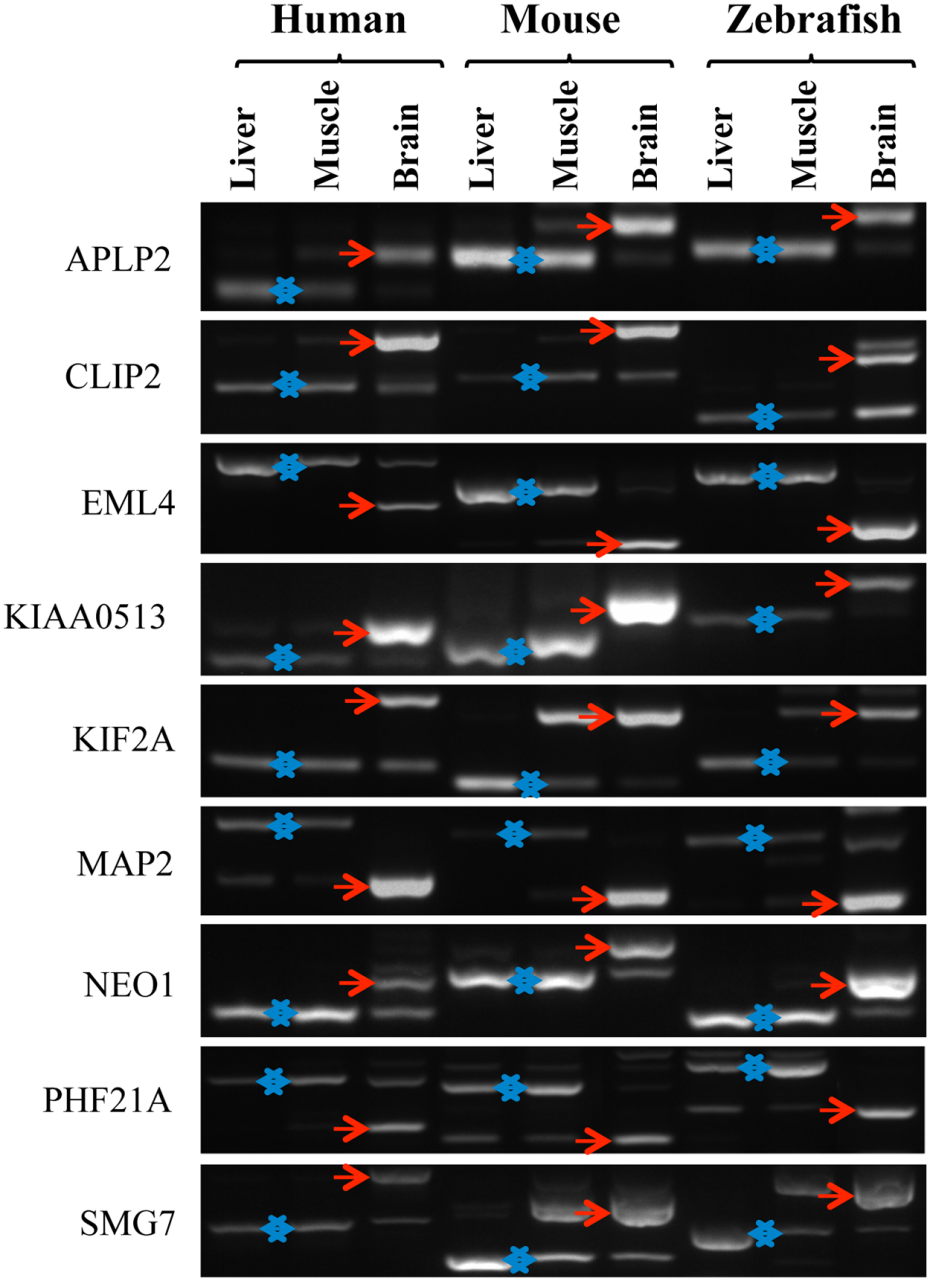
Switch-like tissue-specific ASEs are conserved in all vertebrates. RT-PCR was performed on 15 genes across human, mouse and zebrafish. The 9 genes shown have conserved switch-like splicing in all three vertebrate species. Brain-specific splice forms are indicated with a red arrow. The alternative kidney/liver-specific forms are indicated with a double-headed blue arrow. The different, expected and found, PCR sizes for the long and short form of each gene in each species are given in S1 Table.

**Fig 4:**
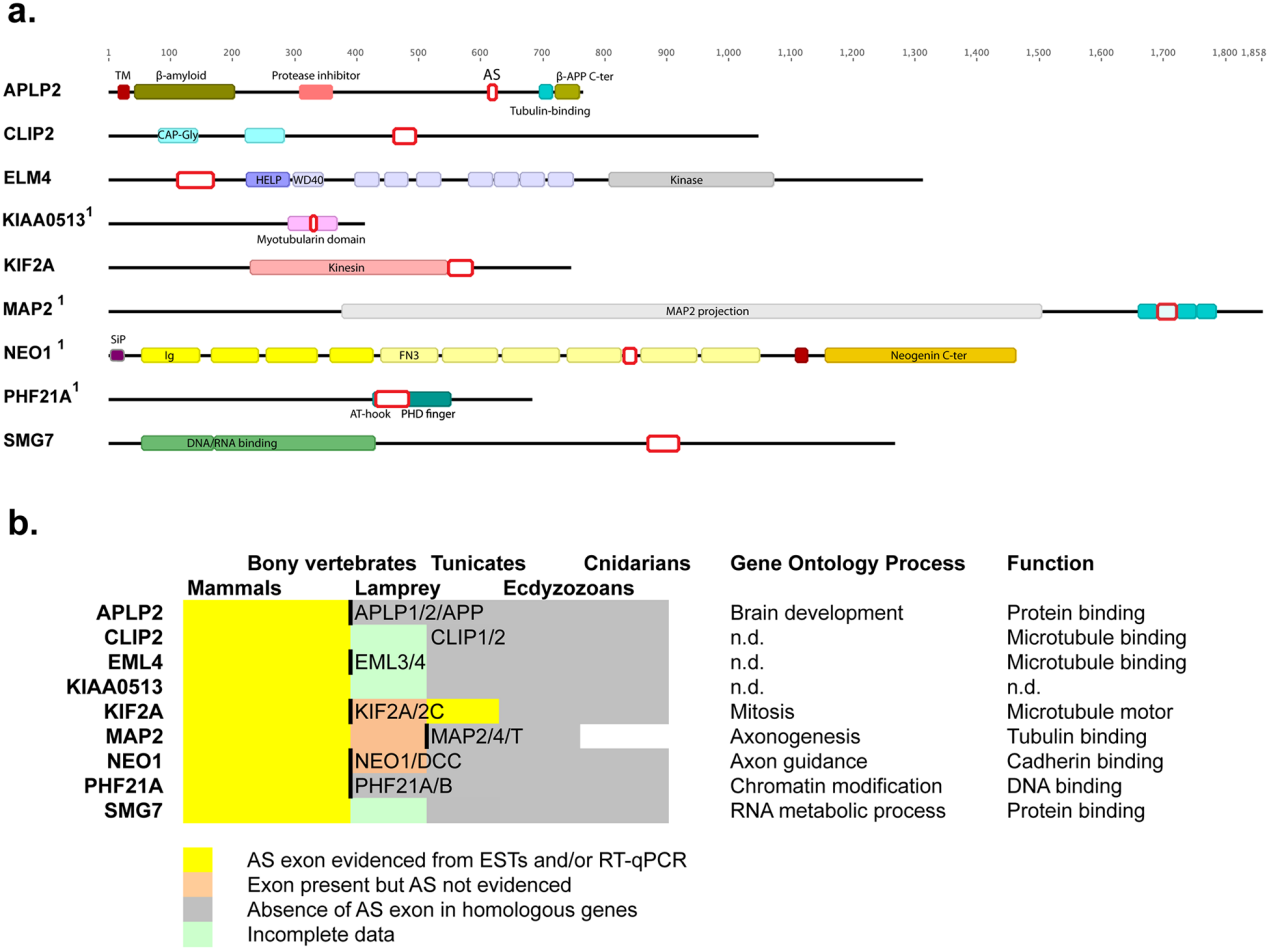
Nine vertebrate brain specific alternative splice events. **a. Primary structures of proteins encoded by the 9 human genes with vertebrate conserved brain-specific splicing.** Shown are the annotated human proteins with the regions of the nine splice events indicated by red boxes. TM: transmembrane region; β-APP C-ter: C-terminus of the β Amyloid Precursor Protein (pf10515); CAP-Gly: Cytoskeletal-Associated protein (pf00225); HELP: Hydrophibic EMAP-Like Protein (pf03451); WD40: β-transducin repeat (pf00400); SiP: Signal peptide; Ig: Immunoglobulin-like domain (pf00047); FN3: Fibronectin type III domain (pf00041); AT-hook: DNA-binding for A/T-rich regions (pf02178); PHD finger: Plant HomeoDomain (Cys)4-His-(Cys)3 (pf00628). Gene names are labelled with a ^1^ if exclusion of the alternatively splice regions directly affects structural domains. Note all ASEs are multiples of 3 nucleotides, thus all the alternative splicing events confer in frame peptide omission or insertion. **b. Brain-specific alternative splicing is conserved in vertebrates, and possibly beyond, in microtubule-associated genes.** Metazoan genomes in Ensembl were searched for paralogs and orthologs of each target gene and for the presence (yellow) or absence (gray) of potential ASEs (Accession numbers are shown in S1 Table). Yellow indicates that alternatively spliced mRNAs were detected in EST databases. Orange indicates that there were too few ESTs to conclude. Green indicates the absence of both genomic and EST data. Duplications are indicated by thick lines along with the names of the duplicated genes. An indication of the function of each gene is given on the right.

To test the conservation of the splicing patterns of these alternative events between mouse and humans, primers were designed to the 89 orthologous human ASEs, we could find, of the 93 brain-regulated mouse ASEs. Splicing profiles were then interrogated by RT-PCRs in six human tissues, brain, liver, kidney, lung, muscle and heart. From these, 62 ASEs produced robust data in all tissues (Fig 2). Combining the percent-spliced-in data for these 62 ASEs from mouse and human (from Figs 1 and 2 respectively) clustered human and mouse tissues together, demonstrating that splicing of this group of ASEs is tissue-specific rather than species-specific (S2 Fig). Furthermore, of all the tissue-specific ASEs that showed similar splicing patterns in mouse and humans, 15 ASEs had a shift of more than 50% in splicing between brain and all of the other tissues. The conservation of the switch-like brain-specific splicing of these 15 ASEs was then further investigated in zebrafish brain, kidney and liver, and 9 of the ASEs were confirmed to be brain-specific in zebrafish: EML4, KIF2A, MAP2, APLP2, NEO1, CLIP2, SMG7, PHF21A and KIAA0513 (Fig 3). We mapped the location of these 9 ASEs onto their respective protein structures, and found exons encoding both known structural domains and inter-domain regions (illustrated diagrammatically in Fig 4a).

Next, we investigated the extent to which the vertebrate brain-specific splicing process might be conserved, as alternative spliced regions, further in evolution than bony vertebrates. The analysis of EST databases by peptide homology searching revealed alternative splicing in genes for NEO1, MAP2 and KIF2A in jawless vertebrates and the latter was also conserved in tunicates, the sister taxon of vertebrates (Fig 4b).

### The conserved splices are dynamically regulated in diverse physiological contexts from man to fish

**Fig 5:**
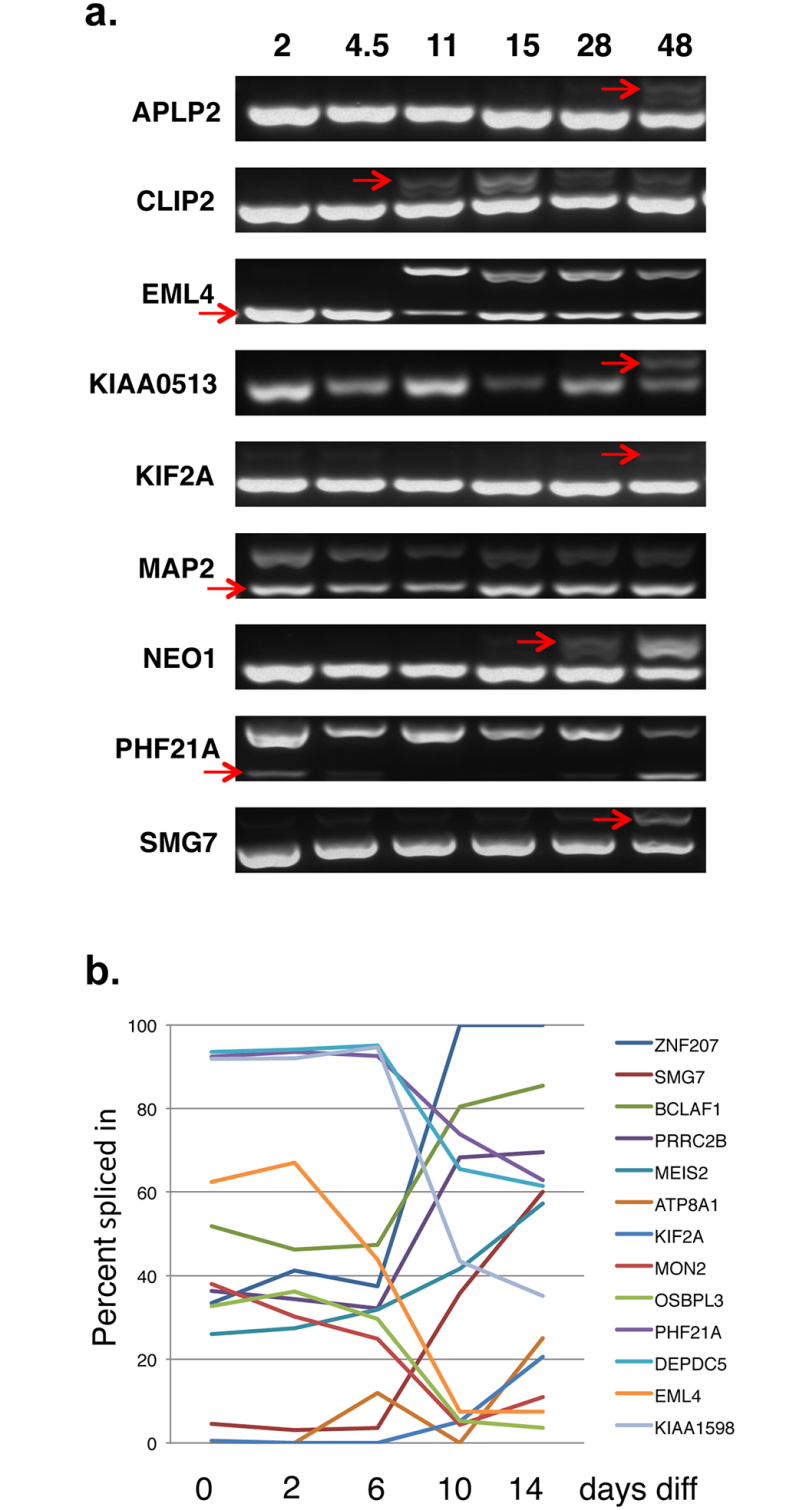
Neural splicing dynamics. **a. The 9 conserved brain-specific ASEs shifts during zebrafish embryogenesis.** Time-course of splicing of the nine vertebrate brain-specific ASEs during the first two days of zebrafish embryogenesis, spanning 2 to 48 hours post fertilisation (hpf). The red arrows indicate the commencement of expression of the brain-specific AS transcript. **b. Splicing of 13 genes pivots from predominantly one AS transcript isoform to the other upon neural differentiation of stem cells between days 6–10.** 93 ASEs were assayed by PCR and their psi values were evaluated during neural stem cell differentiation. 56 ASEs gave good data for all time points (see S1 Table). Chart showing the psi values of 13 ASEs whose psi values shift more than 50% during the time-course.

To understand how alternative splicing of these conserved ASEs might be modulated within an intact organism, we monitored them during the first 48 hours of embryogenic zebrafish development (Fig 5a). Each showed a dynamic pattern over this time frame. If these brain-specific splicing events are important for brain, they would also be expected to change during the course of neural stem cell differentiation. To test this hypothesis, we turned to an in vitro differentiation system (N1997 cells) that converts stem cells to a mixture of neurones, astrocytes and oligodendrocytes [11,12]. We tested all 89 mammalian brain-specific ASEs and observed a sharp change at 6–10 days of differentiation with 13 ASEs shifting over 50% (i.e. in a switch-like manner) in one direction, and all at or around this time point (Fig 5b).

### Conserved alternative splicing targets the cytoskeleton

The gene encoding Echinoderm Microtubule-Associated Protein-Like 4 (EML4), which affects the assembly dynamics of microtubules [13], was identified as undergoing among the largest shifts in both human stem cell differentiation and during zebrafish embryogenesis. Therefore we investigated the effect of splicing of EML4 at the protein level by Western blotting and confirmed a shift from the exon-included to the exon-skipped form of EML4 protein during stem cell differentiation (S3 Fig).

As over half of the evolutionarily conserved splicing changes identified in this study are involved in microtubule and or cytoskeleton dynamics we tested a panel of microtubule-related genes for their tissue-specific splicing patterns. The strongest shifts were displayed by EML1 (a paralog of EML4) and KIF13A, which is a gene encoding a kinesin microtubule-based motor, like KIF2A (S4 Fig). Another kinesin, KIF1b had already been shown to be alternatively spliced in brain [14,15] and it is regulated along with KIF2A and KIF5A by PTBP1 which is down-regulated in brain [16–18]. We thus analysed the splicing of 65 exons in 29 kinesin genes (S1 Table and http://rnomics.med.usherbrooke.ca:3000/data/related/2149). Just 17 ASEs gave good data in all six tissue tested. This analysis confirmed brain-specific splicing of KIF2A and KIF13B.

## Discussion

In this study, we identify a panel of alternative splice events (ASEs) that switch dramatically in their inclusion levels between brain and other tissues of three distantly related vertebrate species: human, mouse and zebrafish. The brain has long been known to have a highly complex transcriptome due to alternative splicing [19] but the conservation of regulation of these ASEs has not been thoroughly investigated. Early indications, from microarray technology, indicated that even in our closest cousins the chimpanzees, there was relatively little conservation of actual observed alternative splicing patterns, even though the genomic regions encoding ASEs were near identical [20]. Many studies, therefore, have documented brain-specific ASEs in one species or another but ours is the first study to verify a group of such switch-like [8] ASEs across vertebrate taxa. Previous studies comparing ASEs across taxa found relatively few brain-specific ASEs [6,21,22], however two ASEs, in the NEO1 and APLP2 genes, have previously been confirmed to be have conserved switch-like brain-specific alternative splicing in vertebrates [21]. Our study confirmed these two switch-like vertebrate brain-specific splicing events but also adds seven others (Fig 3). These brain-specific splices are likely of importance for neural biology as four of them: NEO1, KIF2A, APLP2, and MAP2 were shown to be alternatively spliced during neuronal differentiation of P19 mouse embryonal carcinoma cells [23] and the latter three ASEs were also shown to be targets of PTBP1, which is a key splicing factor that is down-regulated during neurogenesis [16,17].

Several of the affected genes have functions in the cytoskeleton and they are particularly associated with microtubules. Microtubule-associated proteins act as important regulators in the staged development of neurite, axon and dendrite formation, and the appearance of alternative splicing of these genes following neural stem cell differentiation indicates that these are key events in these processes [24]. These results also support the hypothesis that the main function of alternative splicing is to enact a program of changes in the cytoskeleton between different cell types [25–27]. The microtubule-associated genes with conserved brain-specific splicing are EML4 (echinoderm microtubule associated protein like 4), MAP2 (microtubule-associated protein 2), KIF2A (microtubule-depolymerizing kinesin) [28] and CLIP2 which associates with the ends of growing microtubules [29]. Furthermore, APLP2 is involved in actin dynamics [30] and KIAA0513 interacts with the cytoskeleton [31]. Conservation analysis indicated that alternative exons of two of the microtubule-associated genes (MAP2, KIF2A) are even more ancient than vertebrate radiation (Fig 4b) and therefore regulation of their splicing may be an important ancient feature of sophisticated neural circuitry in deuterostomes. These alternatively spliced genes also have important functions in the brain. Although mice lacking MAP2 were normal, combined knockdown of MAP2 and MAP1B led to dendritic outgrowth problems [32]. KIF2A knockdown mice show aberrant axonal branching [33]. CLIP2 knockout mice have hippocampal dysfunction and motor coordination defects [34].

Five of the nine conserved ASEs (EML4, CLIP2 SMG7, PHF21A, KIAA0513) have not previously been reported to be alternatively spliced in any species’ brain. The ASE detected in the gene for the microtubule-associated protein EML4 was the most strongly altered event in all our validation assays. EML4 splicing shifts nearly completely during zebrafish embryogenesis (Fig 5a) as well as in stem cell differentiation (Fig 5b) where we confirmed the change at the protein level (S3 Fig). EML4 is currently under intense investigation in non small cell lung cancer where it is frequently found fused to the ALK gene [35]. In a further screen of microtubule-associated splice events, we also found brain-specific splicing of its homologue EML1 and of, another kinesin microtubule motor protein gene, KIF13A (S4 Fig). We previously found KIF13A exon inclusion during stem cell differentiation to primary mesoderm under the control of the MBNL1 and RBFOX2 splicing factors [2]. As all our observed ultra-conserved splicing changes are in-frame events (Fig 4a), this is consistent with the idea that the function of alternative splicing is to regulate protein interaction networks by the removal and addition of protein interaction domains [36] which might be especially important during the complex cytoskeletal changes occurring during neurogenesis [24]. To conclude, our study reveals the existence of at least 9 vertebrate-conserved switch-like brain-specific ASEs, which very likely have a direct bearing on the establishment of the neuronal microtubule network.

## Methods

### Ethics statement

Zebrafish embryogenesis was specifically approved by the Direction Sanitaire et Vétérinaire de l’Hérault and Comité d’Ethique pour l’Expérimentation Animale under reference CEEA-LR-13007. Zebrafish were handled according to European Union guidelines for the handling of laboratory animals (http://ec.europa.eu/environment/chemicals/lab_animals/home_en.htm). Adult mice were sacrificed by carbon dioxide inhalation as per standard operating procedures approved by the IRCM ACC and the CCAC. All efforts were made to minimize the number of animals used and to reduce their suffering. The protocols for the mice used in this study were reviewed and specifically approved by the IRCM Animal Care Committee (ACC, protocol number: 2009–12). Mice were sacrificed and organs were prepared according to institutional rules put in place by the IRCM Animal Care Committee (ACC Quebec Comité de protection des animaux), which follows the regulations and requirements of the Canadian Council on Animal Care (CCAC, www.ccac.ca)

### Animals

Fish maintenance, staging and husbandry were as described previously [37] and performed under standard conditions [38]. The F1 golden zebrafish mutant [39] originally purchased from Antinea (http://groupe-antinea.fr/) was used. Embryos were obtained from pairs of adult fish by natural spawning and raised at 28.5°C. Embryos and larvae were staged as described previously [40]. Mice were housed under specific pathogen-free conditions and certified animal technicians regularly observed the mice. All mice were from the C57BL/6 genetic background and were offspring from animals originally obtained from The Jackson Laboratory (Bar Harbor, Maine, USA).

#### High-throughput splicing experiments

were performed as in [3]. For analysis of splicing data, a quality control was used whereby PCRs were only considered with purity >75%, that is with >75% of the total molarity of the found peaks, found at the expected molecular weights. Percent spliced in (psi) values were calculated as the molarity of the long form divided by the molarity of the long and short combined. Manual PCRs and electrophoresis were performed as in [2]. All data is visible on our website http://rnomics.med.usherbrooke.ca:3000/pcrreactiongroup/list/336. Each section contains clickable graphics which leads to the primary electrophoresis data. Links are also available to download all the calculated splicing data in Excel format.

#### Human neuronal cells

were generated from the human neuronal precursor stem cell (hNPSC) line N1997 [11,12]. The hNPSCs were seeded onto geltrex-coated cell culture plates in proliferation media (Advanced Dulbecco’s Modified Eagle’s Medium F12—Life Technologies) supplemented with 1% N-2 solution (Life Technologies), 2% B27 solution (Life Technologies), 1% ITS (10 μg ml^-1^ insulin, 5.5 μg ml^-1^ transferrin, 5 ng ml^-1^ sodium selenite solution), 1% PSG (100 units ml^-1^ penicillin, 100 μg ml^-1^ streptomycin, 2 mM glutamine), 2.5 μg ml^-1^ fungizone (Amphotericin B, Life Technologies), 1% MEM non-essential amino acids (100 X, Life Technologies), 2% sodium bicarbonate (7.5% solution), 1.5% D-glucose (45% in H_2_O), 20 mM L-ascorbic acid, 5 μg ml^-1^ heparin, 20 ng ml^-1^ epidermal growth factor (EGF, Sigma Aldrich), 20 ng ml^-1^ basic fibroblast growth factor (FGF2, R and D Systems) and 10 ng ml^-1^ leukaemia inhibitory factor (LIF, Sigma Aldrich). The hNPSC were cultured in proliferation media for 4 days prior to the start of differentiation into mature neural cells. To induce terminal differentiation, the proliferation media was withdrawn and replaced with differentiation media, which was prepared as the proliferation media but without the addition of EGF, FGF2 and LIF and with the addition of 10% fetal bovine serum (Sigma Aldrich). The hNPSCs were differentiated for 14 days with differentiation media being refreshed every other day. At days 0, 2, 6, 10 and 14 of differentiation, the cells were harvested for PCR by the addition of 500 μl of Trizol reagent (Sigma Aldrich) per well, after which the cells were scraped from the surface and collected. The protein samples for western blotting were collected by washing the cells with Dulbecco’s phosphate buffered saline prior to lysis with native lysis buffer.

#### Western blotting

was performed by the addition of 10 μg of protein from the stem cell lysates, determined by a modified Bradford assay, to the lanes of 4–12% Bis-Tris Midi gels (Life Technologies). The proteins were separated by electrophoresis, then transferred to a nitrocellulose membrane using the iBlot gel transfer system (Life Technologies). The nitrocellulose membrane was blocked with tris-buffered saline with 0.05% TWEEN 20 and 5% w/v non-fat dried skimmed milk, prior to probing with primary antibodies EML4 (HPA036688 Sigma Aldrich) and GAPDH, labelled with HRP (sc-25778 HRP Santa-Cruz), all at 1:4000 dilutions. The anti-EML4 antibody was detected with sheep anti-rabbit antibody, labelled with HRP (ab6795 Abcam). The substrate ECL 2 (Fisher Scientific) was added, and the membranes were exposed to FujiFilm Super RX films (Fisher Scientific) prior to development in Kodak GBX developer and fixer solutions.

#### Conservation analysis

was performed as in [2]. Orthologous genes and exons corresponding to alternatively spliced variants were searched at http://ensembl.org (vertebrates, tunicates, *D*. *melanogaster* and *C*. *elegans*) and at http://www.metazome.net/ (cnidarians). Splicing variants were identified in EST databases (http://blast.ncbi.nlm.nih.gov/). Accession numbers are in S1 Table.

## Supporting Information

S1 Fig High-throughput RT-PCR shows brain has the most distinct splicing profile amongst six human tissues . PCR was performed across regions of alternative splicing in 47 diverse genes on 6 human tissue cDNA libraries. The percent-spliced-in (psi) values were calculated and plotted as a heat map. The psi values are indicated by a colour going from yellow to blue depending on the extent to which the alternative exon is spliced in for that tissue. The six tissues were clustered, based on their psi values, which shows brain has the most distinct splicing profile in human.
(TIF)

S2 Fig A group of mammalian tissue-specific ASEs. Data from human ASEs in Fig 2 was combined with data for the orthologous mouse ASEs from Fig 1. The psi values are shown in the heat map; the genes were ordered according to the shift between human brain and the closest of the other five human tissues. Note that brain clusters separately from the other tissues, irrespective of species. Note also, the 4 top-most and 11 bottom-most ASEs psi values shift more than 50% between human brain and the other five tissues.
(TIF)

S3 Fig Splicing shifts to the short form of the EML4 protein during stem cell differentiation. A. Representative manual PCRs for the most significant shifts in either direction. Times are shown in days. B. Western blot showing EML4 expression during stem cell differentiation. Biological triplicate samples were probed with anti-EML4 and anti-GAPDH antibody as a loading control. Note the shift in splicing predicted by the PCR experiments is verified at the protein level. Note also, a shorter unidentified protein is visible in all samples except the fully matured 14 day samples.
(TIF)

S4 Fig Brain-specific splicing of microtubule-associated genes. 94 exons in 74 microtubule-associated genes were assayed in 6 human tissues. Heat-map showing percent-spliced-in values for 39 alternative exons that gave good data for all six tissues. The genes were clustered, depending on their psi values patterns across the 6 tissues. Note, brain showed the most significant tissue-specific splicing.
(TIF)

S1 Table **Tab1.** Splicing data for cross-evolutionary comparison. All data superior to the 75% purity cut off (see methods) in all tissues is shown. The nine ultra-conserved regulated human ASEs are shown in red. Column **a.** Human ASE names (gene names followed by a letter if more than one ASE occurs in this gene). **b.** Mouse gene name. **c.** Mouse ASE name (gene names followed by the ASE size if more than one ASE occurs in this gene). **d.** ASE type. **e.** Mouse in-house primer names separated by a double space. **f,g.** Mouse forward and reverse primer sequences. **h.** Mouse upstream intron sequence (150ntds). **i.** ASE sequence. **j.** Mouse downstream intron sequence 150 ntds. **k-m**. Mouse small and large PCR product and ASE sizes (l-k) respectively. **n-p.** Mouse brain, kidney and liver psi values respectively. **q.** Shift between brain psi and the nearest of liver or kidney. **r.** Human gene name again. **s.** Human in-house primer names separated by a double space. **t,u.** Human forward and reverse primer sequences. **v-x.** human small and large PCR product and ASE sizes (w-v) respectively. **y-ad.** Human brain, kidney, liver, lung, muscle and heart psi values respectively. **ae-ai.** Human neural stem cell psi values at 0, 2, 6, 10 and 14 days of differentiation. **aj,ak.** Zebrafish forward and reverse primer sequences. **al,am.** Zebrafish small and large PCR product sizes. **Tab2. Splicing data for microtubule-related genes (rows 2–44) and Kif genes (rows 45–58). a.** Gene name **b,c.** Short and long PCR product sizes. **d.** Alternative splice event type. **e.** In-house primer names. **f,g.** Forward and reverse primer sequences. **h-m.** Percent-spliced-in values in the indicated tissues. **Tab3. Accession numbers for evolutionary analysis of alternative splice events.**
(XLS)
