# Supplementary figures and images for: Neural Differentiation Modulates the Vertebrate Brain Specific Splicing Program
Source: PLoS One. 2015 May 19;10(5):e0125998. doi: 10.1371/journal.pone.0125998 (PMC4438066; doi:10.1371/journal.pone.0125998)

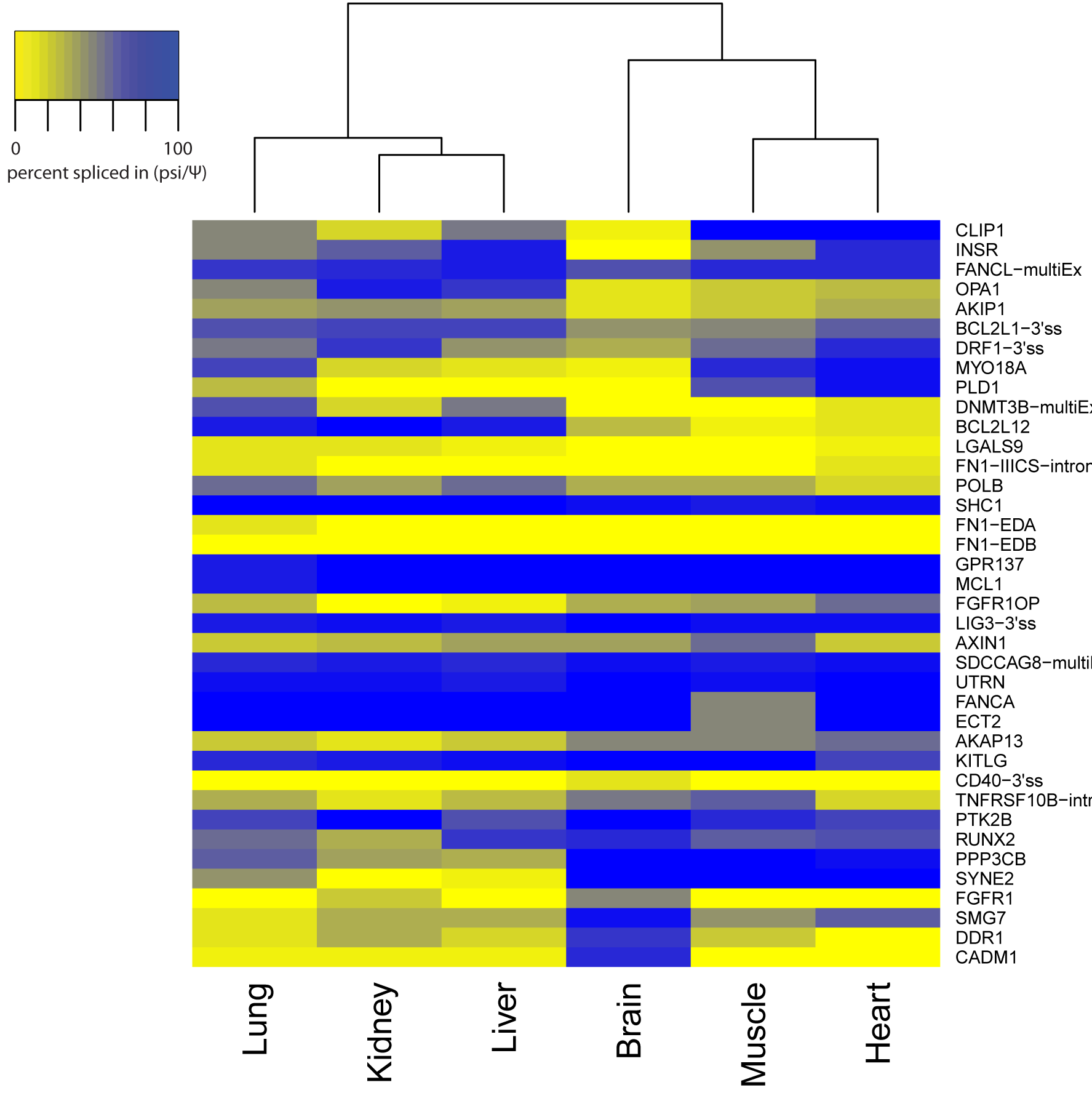

Supplement: S1 Fig — . PCR was performed across regions of alternative splicing in 47 diverse genes on 6 human tissue cDNA libraries. The percent-spliced-in (psi) values were calculated and plotted as a heat map. The psi values are indicated by a colour going from yellow to blue depending on the extent to which the alternative exon is spliced in for that tissue. The six tissues were clustered, based on their psi values, which shows brain has the most distinct splicing profile in human. (TIF) [file pone.0125998.s001.tif]

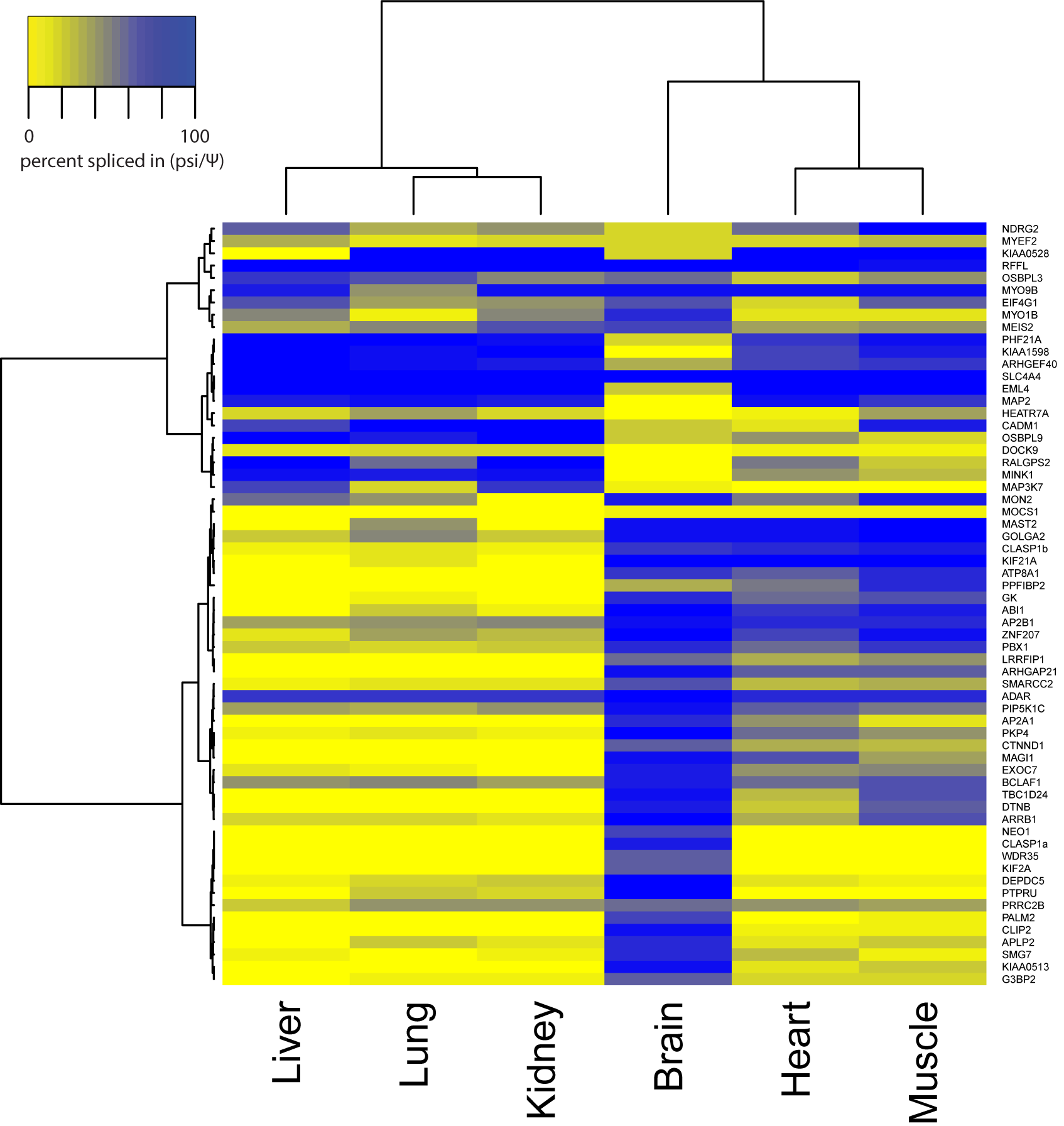

Supplement: S2 Fig — Data from human ASEs in Fig 2 was combined with data for the orthologous mouse ASEs from Fig 1. The psi values are shown in the heat map; the genes were ordered according to the shift between human brain and the closest of the other five human tissues. Note that brain clusters separately from the other tissues, irrespective of species. Note also, the 4 top-most and 11 bottom-most ASEs psi values shift more than 50% between human brain and the other five tissues. (TIF) [file pone.0125998.s002.tif]

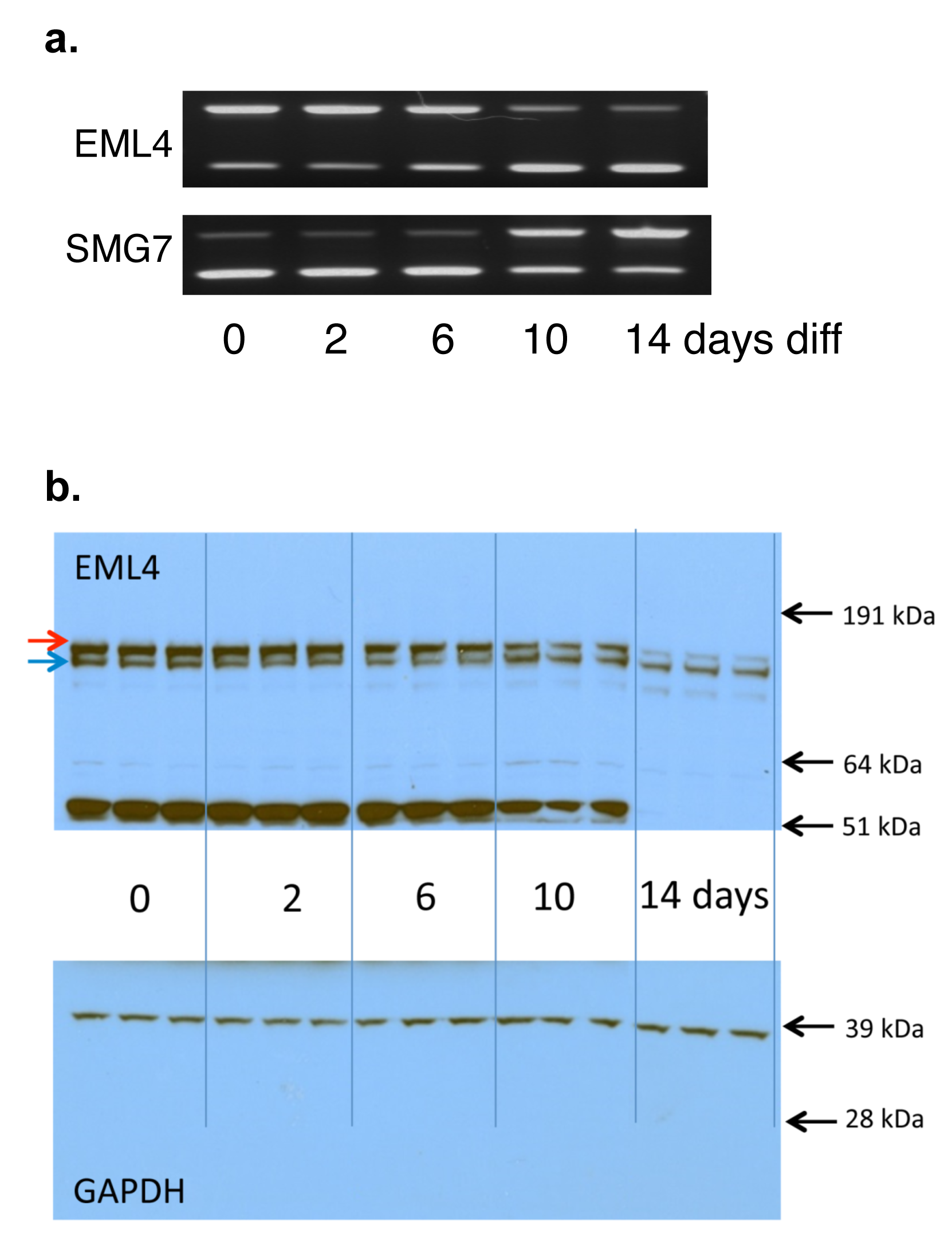

Supplement: S3 Fig — A. Representative manual PCRs for the most significant shifts in either direction. Times are shown in days. B. Western blot showing EML4 expression during stem cell differentiation. Biological triplicate samples were probed with anti-EML4 and anti-GAPDH antibody as a loading control. Note the shift in splicing predicted by the PCR experiments is verified at the protein level. Note also, a shorter unidentified protein is visible in all samples except the fully matured 14 day samples. (TIF) [file pone.0125998.s003.tif]

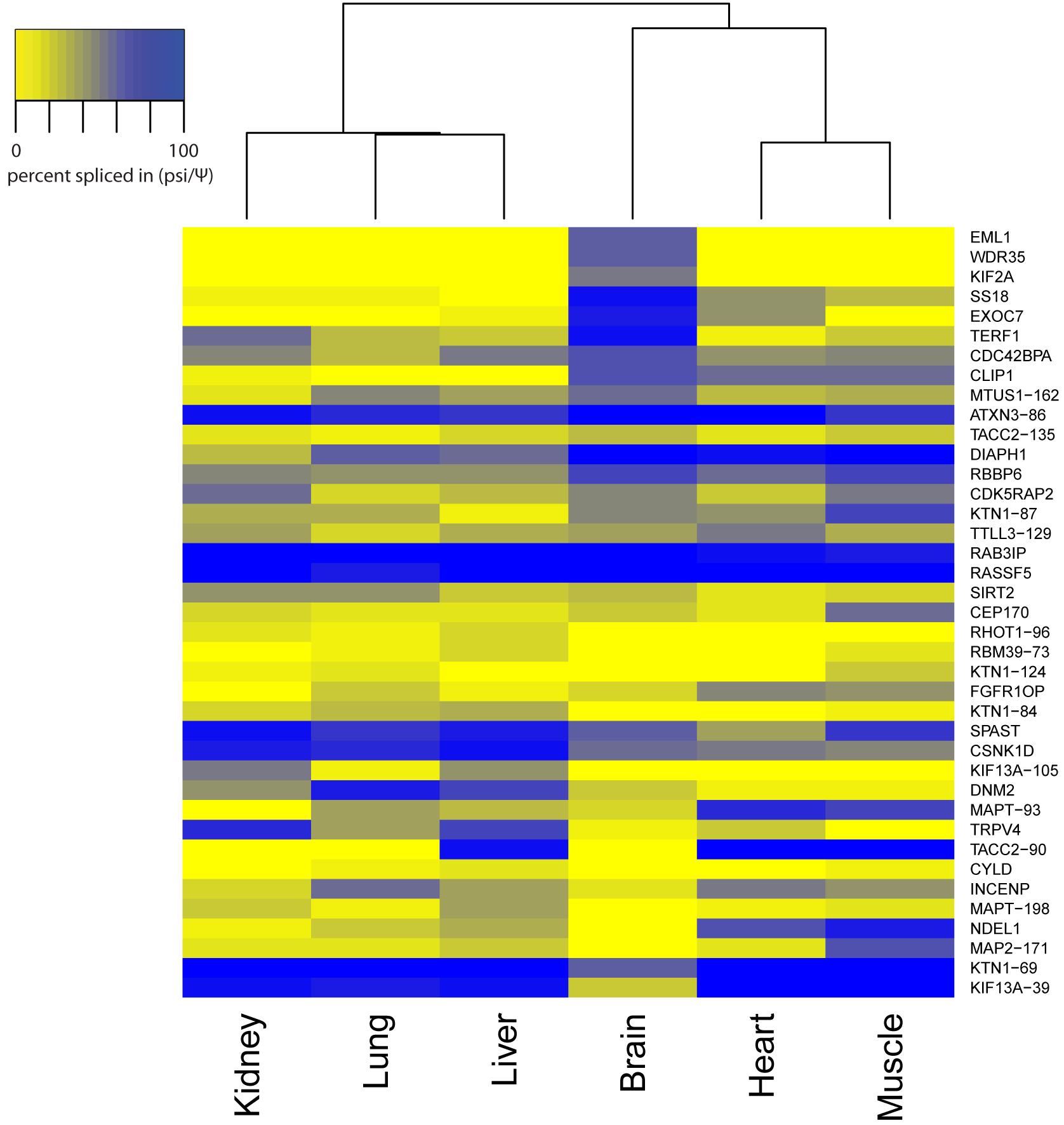

Supplement: S4 Fig — 94 exons in 74 microtubule-associated genes were assayed in 6 human tissues. Heat-map showing percent-spliced-in values for 39 alternative exons that gave good data for all six tissues. The genes were clustered, depending on their psi values patterns across the 6 tissues. Note, brain showed the most significant tissue-specific splicing. (TIF) [file pone.0125998.s004.tif]
